# Prevalence and Factors Associated with Mental Health Impact of COVID-19 Pandemic in Bangladesh: A Survey-Based Cross-Sectional Study

**DOI:** 10.5334/aogh.3269

**Published:** 2021-04-26

**Authors:** Tanvir Abir, Nazmul Ahsan Kalimullah, Uchechukwu Levi Osuagwu, Dewan Muhammad Nur–A Yazdani, Taha Husain, Piwuna Christopher Goson, Palash Basak, Md Adnan Rahman, Abdullah Al Mamun, P. Yukthamarani Permarupan, Md Yusuf Hossein Khan, Abul Hasnat Milton, Kingsley E. Agho

**Affiliations:** 1 College of Business Administration (CBA), IUBAT-International University of Business, Agriculture and Technology, Dhaka, Bangladesh; 2 Begum Rokeya University, Rangpur-5404, Bangladesh; 3 Diabetes, Obesity and Metabolism Translational Research Unit, Western Sydney University, Campbelltown, NSW 2560, Australia; African Vision Research Institute (AVRI), University of KwaZulu-Natal, Westville Campus, Durban, 3629, South Africa; 4 College of Business Administration (CBA), IUBAT-International University of Business, Agriculture and Technology, Dhaka, Bangladesh; 5 Department of Gender and Development Studies, Begum Rokeya University, Rangpur 5404, Bangladesh; 6 Department of Psychiatry, College of Health Sciences, University of Jos, Nigeria; 7 School of Environment and Life Sciences (Environmental Science and Management), University of Newcastle, Callaghan 2308, Australia; 8 College of Business Administration (CBA), IUBAT-International University of Business Agriculture and Technology, Dhaka, Bangladesh; 9 Faculty of Business and Management, UCSI University, Kuala Lumpur 56000, Malaysia; 10 Faculty of Entrepreneurship and Business, Universiti Malaysia Kelantan, Kota Bharu 16100, Malaysia; 11 College of Tourism and Hospitality Management, IUBAT-International University of Business Agriculture and Technology, Dhaka, Bangladesh; 12 Research International, Dhaka, Bangladesh & Epidemiology Resource Centre, NSW, Australia; 13 School of Health Science, Western Sydney University, Campbelltown, NSW 2560, Australia; African Vision Research Institute (AVRI), University of KwaZulu-Natal, Westville Campus, Durban, 3629, South Africa

## Abstract

**Background:**

Feelings of isolation, insecurity, and instability triggered by COVID-19 could have a long-term impact on the mental health status of individuals.

**Objectives:**

The aim of this study was to examine the prevalence of mental health symptoms (anxiety, depression, and stress) in Bangladesh and the factors associated with these symptoms during the COVID-19 pandemic.

**Methods:**

From 1 to 30 April 2020, we used a validated self-administered questionnaire to conduct a cross-sectional study on 10,609 participants through an online survey platform. We assessed mental health status using the Depression, Anxiety, and Stress Scale (DASS-21). The total depression, anxiety, and stress subscale scores were divided into normal, mild, moderate, severe, and multinomial logistic regression was used to examine associated factors.

**Findings:**

The prevalence of depressive symptoms was 15%, 34%, and 15% for mild, moderate, and severe depressive symptoms, respectively. The prevalence of anxiety symptoms was 59% for severe anxiety symptoms, 14% for moderate anxiety symptoms, and 14% for mild anxiety symptoms, while the prevalence for stress levels were 16% for severe stress level, 22% for moderate stress level, and 13% for mild stress level. Multivariate analyses revealed that the most consistent factors associated with mild, moderate, and severe of the three mental health subscales (depression, anxiety, and stress) were respondents who lived in Dhaka and Rangpur division, females, those who self-quarantined in the previous seven days before the survey, and those respondents who experienced chills, breathing difficulty, dizziness, and sore throat.

**Conclusion:**

Our results showed that about 64%, 87%, and 61% of the respondents in Bangladesh reported high levels of depression, anxiety, and stress, respectively. There is a need for mental health support targeting women and those who self-quarantined or lived in Dhaka and Rangpur during the pandemic.

## Introduction

As the global population tries to make sense of the transformations, including personal adjustments to lifestyle, brought about by the COVID-19 pandemic, residents of low- to middle-income countries (LMIC) including Bangladesh face greater challenges due to the fragile health systems [1,2], the dense population of Bangladesh, and the fact that the country houses a million stateless Rohingya refugees in sprawling refugee camps that are conducive to the spread of epidemics. Bangladesh also has significant migrant populations living in Italy, a COVID-affected country [1]. Whilst the mortality rates in Bangladesh have remained low, due to the timing of the infection, the early transmission of the virus, and the response to the pandemic by authorities, the low socio-economic status of the country and the existing health inequalities usually lead to worse effects [3].

Science has played a significant role in improving people’s understanding of the virus, finding effective ways of containment through timely sequencing of the virus and rapid sharing of the data [4], and most recently the development of different vaccines. Unraveling the genetic sequence of the SARS-COV-2 virus about four weeks after the outbreak of the SARS-COV-2 virus [5] was short compared to the Spanish flu, which took almost seven decades for scientists to unravel the genetic sequence of the disease [6], and was crucial to the development of a diagnostic test and potential treatment [7,8]. Globally, the virus has infected over 84 million people, including 1.8 million reported deaths from the infection at the time of this study. In Bangladesh, there have been 515,000 confirmed cases as of 2 January 2021, with 7,576 deaths reported to the World Health Organization (WHO) [9]. The COVID-19 pandemic spread faster, and the mortality rate is higher, than those attributed to the Middle Eastern respiratory syndrome (MERS) and Severe acute respiratory syndrome (SARS). Thus, there was fear and panic among residents as news about its fatal nature spread very easily through traditional and social media outlets, leaving behind a trail of despair and disruptions in lifestyle. The high mortality rate, closure of businesses, and strict containment measures by national governments also added to the hidden and unhidden mental health burden of the pandemic [10,11]. However, no one has been able to report comprehensively the mental health impact of the pandemic in most LMIC.

Despite the delay in COVID-19 cases in Bangladesh (the first case was reported 18 March 2020), the country’s global supply chain of international fashion brands and human resource exports suffered a huge set-back with devastating psychosocial consequences emanating from the international and local economic impacts [12]. Post-traumatic stress symptoms, as well as delayed grief and the sense of loss, after multiple deaths and loss of jobs and avenues to socialize have been reported in previous studies [13].

The burden of mental disorders is already high in Bangladesh, and this is a largely unrecognized and under-researched area in Bangladesh as shown in a review study [14]. In addition to the mental health burden from displaced Rohingya refugees [15], the outbreak of COVID-19 may have an additional negative impact on the state of mental health in Bangladesh. In a web-based cross-sectional study conducted in the US using electronic Qualtrics software, female college students reported higher levels of perceived stress and inability to focus on their academic work during the COVID-19 pandemic [16]. Similarly, in a web-based observational study conducted in Italy, which evaluated the impact of the COVID-19 pandemic on infertile couples’ emotions, anxiety, and future plans, the authors used the Survey Monkey platform and the web link of the survey was sent via emails and published on six online forums frequented by infertile patients. The couples undergoing assisted reproductive treatment showed severe psychological impact of the COVID-19 pandemic, particularly among women who were more emotionally distressed, anxious, and depressed than their men counterparts. This was because assisted reproductive treatment was stopped in many centres due to rising concern as to the impact of COVID-19 on pregnancy [17]. Coupled with these are the uncertainties about effective treatment, availability of effective vaccines, as well as whether life could return to normal.

All these could negatively affect the mental health of the populace and, by extension, the productivity of the country that depends largely on international trading, which has so far been decimated by the pandemic. Health care delivery in Bangladesh has major challenges, including weak governance and an over-centralized framework, the poorly regulated private sector that employs over 58% of all physicians work in the poorly regulated private sector, and the lack of funding for the public sector [18]. Layered on top of these are the lack of resources and the disproportionate distribution of mental health services in Bangladesh, leading to poor access to mental health facilities and care [19]. Therefore, the aim of this study was to determine the prevalence of mental health symptoms during the pandemic and to identify the factors associated with the mental health symptoms in a Bangladesh population. The findings of this study will provide meaningful supplementary and complementary data to inform the understanding of the impact of the COVID-19 pandemic on global mental health regarding a densely populated, geopolitically and economically-critical region.

## Methods

### Study design and setting

A cross-sectional online study was conducted in Khulna, Chittagong, Mymensing, Rajshahi, Barisal, Rangpur, Sylhet, and Dhaka divisions in Bangladesh from April 1–30, 2020, corresponding to the mandatory lockdown period imposed in different parts of the country.

Bangladesh is a developing nation in South-East Asia that became a separate political and economic entity only 50 years ago. Originally part of the British Raj and then pre-Independence India, Bangladesh became an eastern province of Pakistan and gained independence in 1947 following its ‘Great Liberation War’ and was named Bangladesh [20]. Bangladesh covers 147,570 square kilometres and has a population of roughly163 million, with India and Myanmar at its borders and majority are Muslims (89%). About 38.2% of the population residing in urban areas, including the capital Dhaka, which has an estimated population of 21 million people and growing [21]. The country has about 220 psychiatrists and 50 trained clinical psychologists serving the whole nation [20] and has absorbed close to a million refugees from Myanmar in recent years.

### Sampling

Invitations to participate were sent out online through social media platforms (e.g., Facebook, Google Plus, LinkedIn, and Twitter) because traditional face-to-face interviews were not possible due to the lockdown. The use of social media platforms also enabled the researchers to reach target respondents living in different parts of Bangladesh. Residents were also asked to share the e-link with their friends and networks to increase the reach. A snow-ball sampling technique was used for collecting information from participants.

To be eligible for participation in this study, the respondents had to reside in Bangladesh, be able to provide online informed consent, and be 18 years and over at the time of data collection. Informed consent was obtained through an online preamble before the respondents began the questionnaire. The participants were assured of the anonymity and confidentiality of their responses and information provided as well as their freedom of choice of participation. The study was approved by the Institutional Review Board of Dr. Wazed Research and Training Institute, Begum Rokeya University, Rangpur (#BRUR/DWRTI/a.n.004). All procedures were in accordance with the 1964 Helsinki Declaration of Helsinki as revised in Fortaleza.

## Measurements

A web-based self-administered survey created on Google forms was distributed across Bangladesh to reach the target respondents living in different parts of Bangladesh. The respondents clicked the link on the platform and responded to the survey voluntarily. The first part of the survey obtained participants’ demographic information.

The survey was divided into five parts as shown in ***Table 1***: The first part gathered demographic information of the participants, including gender, age, living area (division), level of education, marital status, and working status. The second part was the household factors, which asked about living arrangements and the number living together. The third part included COVID-19 factors, which asked whether/not the participants had been tested for COVID-19. The fourth part evaluated the compliance with WHO recommended precautionary measures, including avoiding crowded gatherings, handshaking and use of public transport, wearing facemasks when going out, advocating with other people about the health risks of the infection. The fifth part evaluated the history of health-related symptoms (if the respondents had experienced any symptoms—fever, pain, headache, chills persistent, dizziness, and breathing difficulties—a couple of weeks before data collection). The information about these five parts is listed in Supplementary Table 1.

**Table 1:** Characteristics of the study population (n = 10,609).


VARIABLES	n	PERCENT (%)

***Demography***		

**Division of Living**		

Khulna	1788	16.85

Chittagong	1198	11.29

Mymensing	288	2.71

Rajshahi	674	6.35

Barisal	716	6.75

Rangpur	464	4.37

Sylhet	221	2.08

Dhaka	5260	49.58

**Gender**		

Male	5238	49.37

Female	5371	50.63

**Level of Education**		

Postgraduate/Post Doctorate	2291	21.59

Graduate	3942	37.16

HSC or Equivalent	2881	27.16

SSC or Equivalent	969	9.13

Under SSC	526	4.96

**Age Category**		

18–27 years	6043	56.96

28–37 years	2423	22.84

38–47 years	1157	10.91

48–57 years	709	6.68

58+ years	277	2.61

**Marital Status**		

Single	4833	45.56

Married	5332	50.26

Divorced/Widowed	444	4.19

**Working Status**		

Working (Full time)	4754	44.81

Working (Part-time)	1220	11.50

Not working/Student	4635	43.69

***Household Factors***		

**Living Arrangement**		

Living with Family	8533	80.43

Living Alone	1504	14.18

Sharing/Living with Flatmates	572	5.39

**Household Size**		

1–2 People	1281	12.07

3–4 People	9328	87.93

**Have you been tested for COVID-19?**		

No	9434	88.92

Yes, I Tested Negative	832	7.84

Yes. I Tested Positive	343	3.23

***Compliance with Public health measures***		

**Have You Enforced Protective Measures Inside Your Home to Protect Yourself and Your Family from COVID 19?**		

Yes	9705	91.48

No	904	8.52

**Are You Currently in Self-Quarantine Since Past Seven Days?**		

No	2155	20.31

Yes	8454	79.69

**What Sort of Protective Measures Have You Taken?**		

Avoid Public Transport		

Yes	2230	21.02

No	8379	78.98

Avoid Shaking Hands		

Yes	2313	21.80

No	8296	78.20

Wearing Face Mask		

Yes	2368	22.32

No	8241	77.68

Avoid Large Gatherings		

Yes	2352	22.17

No	8257	77.83

Advocating People About the Health Risk Related to COVID-19		

Yes	1792	16.89

No	8817	83.11

**Responses Regarding the Health Condition for the Last Couple of Weeks**		

Fever For at least A Day		

No	9179	86.52

Yes	1430	13.48

Chills For at least a Day		

No	9225	86.95

Yes	1384	13.05

Headache For at least a day		

No	7851	74.00

Yes	2758	26.00

Cough For at least a Day		

No	8228	77.56

Yes	2381	22.44

Breathing Difficulty		

No	9207	86.78

Yes	1402	13.22

Dizziness		

No	8802	82.97

Yes	1807	17.03

Sore Throat		

No	8761	82.58

Yes	1848	17.42

Persistent fever and cough or difficulty in breathing		

No	9727	91.69

Yes	882	8.31


### Outcome Measures

The mental health symptoms of depression, anxiety, and stress were the outcome measures. The Depression, Anxiety and Stress Scale-21 Items (DASS-21) is a set of three self-report scales designed to measure the emotional states of depression, anxiety, and stress and were calculated based on a previous study [22]. These instruments are often used to assess subjective complaints and do not directly imply that respondents have a discrete diagnosis as classified in the diagnostic and statistical manual of mental disorders (DSM) and international classification of diseases and related health problems (ICD) [23]. However, their use in this study was predominantly aimed at assessing the perceived severity of symptoms related to depression, anxiety, and stress [24].

### Depression subscale

Responses to each item were rated from 0 (never) to 3 (almost always). Items 3, 5, 10, 13, 16, 17, and 21 (see supplementary Table 1) were classified as the depression subscale, and the total depression subscale score was multiplied by 2 to calculate the final score and then divided into normal (0–9), mild depression (10–13), moderate depression (14–20), severe depression (21+). The validity of the tool was tested, and the Cronbach’s alpha coefficient ranged from 0.75 to 0.77 among the depression subscale, indicating a satisfactory level of reliability.

### Anxiety subscale

Items 2, 4, 7, 9, 15, 19, and 20 (see supplementary Table 1) formed the anxiety subscale, and the total anxiety subscale score was multiplied by 2 to calculate the final score and then divided into normal (0–7), mild anxiety (8–9), moderate anxiety (10–14), severe anxiety (15+) [18]. The Cronbach’s alpha coefficient testing the validity of the anxiety subscale showed scores ranging from 0.65 to 0.77, indicating acceptable internal consistency.

### Stress subscale

Items 1, 6, 8, 11, 12, 14, and 18 (see supplementary Table 1) were classified as the stress subscale. The total stress subscale score was multiplied by 2 to calculate the final score and then divided into normal (0–14), mild stress (15–18), moderate stress (19–25), severe stress (26+), and extremely severe stress (35–42) [22]. The Cronbach’s alpha coefficient of stress subscale scores ranged from 0.78 to 0.81, indicating an acceptable level of internal consistency.

## Data Analysis

### Statistical analysis

Data were analysed using the STATA/MP Version.14.1 (Stata Corp 2015, College Station, TX, USA). Descriptive statistics using frequency tabulations were used to present the sample characteristics. The prevalence and 95% confidence intervals (CIs) for normal, mild, moderate, and severe levels of depression, anxiety, and stress were calculated. The association was further tested by odds ratios (OR) using univariate and multiple multinomial logistic regression analyses to identify factors associated with symptoms of depression, anxiety, and levels of stress. In the multiple multinomial logistic regression analyses, four-stage modeling was employed. In the first stage, the demographic factors were entered into the model. We conducted a manually executed elimination method to determine factors associated with symptoms of depression, anxiety, and levels of stress. The significant factors in the first stage were added to the household factors in the second stage model; this was then followed by the elimination procedure. We used a similar statistical approach for compliance with public health and health condition/factors in the third and fourth stages, respectively. Associations were presented as unadjusted OR for all explanatory variables and then adjusted OR (AOR) with their 95% CI for the variables retained in the final step. The level of statistical significance was set at p < 0.05.

### Spatial analysis

We conducted spatial distribution for the three mental health subscales. A series of maps were prepared using ArcGIS Desktop 10.8 [25]. The average level of depression, anxiety, and stress for the first-level administrative unit of Bangladesh (division) was calculated based on the identification of factors through map comparisons and regression analysis for mild, moderate, and severe levels of the mental health variables. In the maps, the adjusted odds ratios (AOR) for each level was categorized into five quantiles and presented using graduated colour symbols.

## Results

### Sociodemographic characteristics of the study sample

***Table 1*** shows the study sample characteristics. Of the 10,900 participants who completed the questionnaire, data for 10,660 adult respondents from the 8 divisions in Bangladesh who provided responses for the outcome measures of interest were included in the analysis. There were 5,238 males (49.1%), mostly young (18–37 years: 8,466, 79.4%), married people (5,332, 50.3%), who had a university degree or higher (6,233, 58.5%), and many lived in Dhaka division (5,260, 49.6%) with their families (8,533, 80.4%) or with up to 3 people (9,328, 87.9%) in a house and were working full time or part-time (5,974, 56.0%) during the pandemic.

There were also 343 individuals (3.2%) with confirmed cases of COVID-19 and 832 individuals (7.8%) who tested negative to COVID-19. Although majority of the respondents reported that they enforced measures in their homes to protect their families (9,705, 91.5%) and had quarantine experience (8,454, 79.7%), more than two thirds (~78% each) did not comply with the public health advice asking people to avoid public transport, handshaking, large gatherings, and to always wear facemasks when going out. These were put in place to contain the spread of the disease. More than two thirds of the participants had at least one symptom of COVID-19 a couple of weeks before data collection, especially persistent fever and cough (9,727, 91.7%) and difficulty breathing (9,207, 86.8%). Additional demographic and epidemic-related characteristics are presented in ***Table 1***.

### Prevalence of depression, anxiety, and stress symptoms

**Figure 1:**
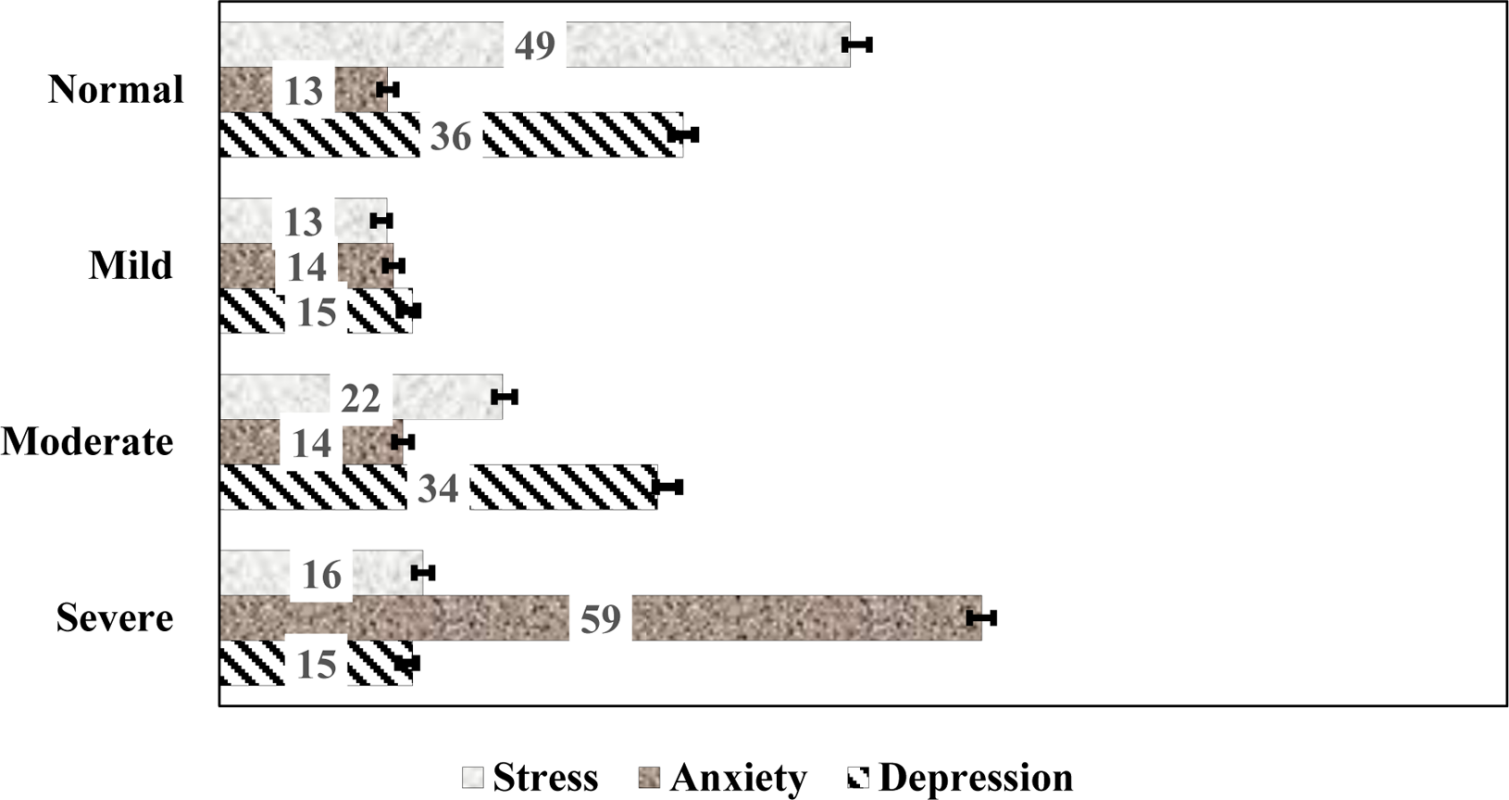
Prevalence of depression, anxiety, and stress levels during the COVID-19 pandemic. Error bars are 95% Confidence Intervals.

The prevalence and 95% CIs for the different levels of the three mental health conditions that were examined in this study are shown in ***Figure 1***. The prevalence was higher for anxiety 86.9% (9,267 participants total, including 1,440 participants [13.5%, 95%CI, 12.4–13.7%] with mild anxiety and 6,310 participants [59.2%, 95%CI, 58.3–60.1%] with severe anxiety), followed by depression 65.0% (6,822 participants total, including 1,563 participants [14.7%, 95%CI, 14.0–15.4%] with mild depression and 1,552 participants [14.6%, 95%CI, 13.9-15.3%] with severe depression), while 50.5% (95% CI, 28.8%–29.6%) reported stress symptoms including severe stress (1,684 participants, 15.8% [95% CI, 15.1–16.5%]).

### Factors associated with symptoms of depression

**Figure 2:**
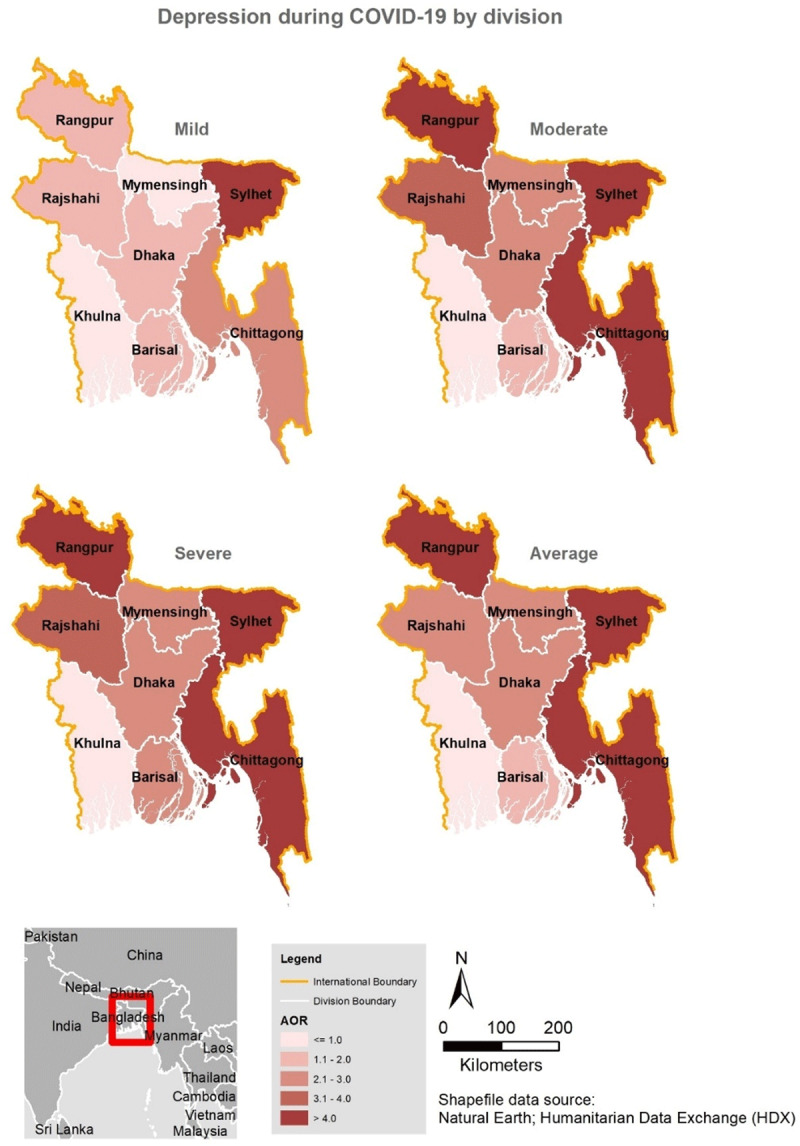
Spatial distribution of depression during COVID-19 in Bangladesh by division.

The results of the unadjusted analysis of demographic and COVID-19 related variables are presented in supplementary Tables 1–3, for depression, anxiety, and stress, respectively. ***Table 2*** presents the multivariate analysis of factors associated with depression in this cohort. In the multivariable analysis, being married; having lower than a postgraduate degree; living alone or living in shared accommodation; living in the Sylhet, Dhaka, Chittagong, Rajshahi, and Rangpur divisions; or having experienced any COVID-19 related health symptoms in the couple of weeks before data collection were associated with symptoms of depression at all levels. ***Figure 2*** presents the adjusted odds ratios for depression symptoms among the respondents by region. The map in ***Figure 2*** indicates that significantly high odds of depression occurred in all eight regions of Bangladesh, but residents in Rangpur, Sylhet, and Chittagong (darkest brown colour) reported the highest odds of severe depression. Individuals who were tested for COVID-19 also had remarkably higher levels of mild–severe symptoms of depression compared to those that had not been tested. In addition, female participants, those who were divorced or separated, residents of Barisal or Mymensing divisions, and those who traveled by public transport displayed a higher odds of moderate–severe symptoms of depression. Individuals with confirmed COVID-19 had at least a 50% higher odds of moderate–severe depression symptoms compared to those not tested for the disease.

**Table 2:** Factors associated with mild, moderate, and severe depression during COVID-19 in Bangladesh.


VARIABLES	MILD			MODERATE			SEVERE	
		
	aOR	P VALUE		aOR	P VALUE		aOR	P VALUE

***Demography***								

**Division of Living**								

Khulna	***1.00***			***1.00***			***1.00***	

Chittagong	2.16 (1.65, 2.81)	<0.001		4.38 (3.47, 5.55)	<0.001		6.38 (4.77, 8.56)	<0.001

Mymensing	0.96 (0.59, 1.57)	0.875		2.68 (1.86, 3.85)	<0.001		2.51 (1.57, 4.01)	<0.001

Rajshahi	1.42 (1.01, 1.98)	0.042		3.56 (2.74, 4.62)	<0.001		3.77 (2.70, 5.27)	<0.001

Barisal	1.04 (0.77, 1.41)	0.782		1.76 (1.36, 2.27)	<0.001		2.09 (1.50, 2.93)	<0.001

Rangpur	1.91 (1.28, 2.86)	0.002		5.07 (3.65, 7.03)	<0.001		5.68 (3.82, 8.45)	<0.001

Sylhet	5.34 (2.93, 9.75)	<0.001		10.67 (6.05, 18.81)	<0.001		15.17 (8.07, 28.53)	<0.001

Dhaka	1.85 (1.54, 2.22)	<0.001		2.48 (2.11, 2.93)	<0.001		2.88 (2.30, 3.62)	<0.001

**Gender**								

Male	***1.00***			***1.00***			***1.00***	

Female	1.14 (0.99, 1.30)	0.060		1.63 (1.45, 1.83)	<0.001		1.71 (1.47, 1.98)	<0.001

**Level of Education**								

Postgraduate/Post Doctorate	***1.00***			***1.00***			***1.00***	

Graduate	1.47 (1.24, 1.75)	<0.001		1.64 (1.41, 1.90)	<0.001		1.55 (1.28, 1.88)	<0.001

HSC or Equivalent	1.25 (1.03, 1.52)	0.026		2.00 (1.70, 2.35)	<0.001		2.19 (1.79, 2.68)	<0.001

SSC or Equivalent	1.53 (1.14, 2.05)	0.005		3.57 (2.84, 4.48)	<0.001		3.22 (2.44, 4.24)	<0.001

Under SSC	1.61 (1.14, 2.28)	0.007		2.64 (1.97, 3.53)	<0.001		2.89 (2.04, 4.10)	<0.001

**Age in category**								

18–27 years	***1.00***			***1.00***			***1.00***	

28–37 years	1.16 (0.94, 1.44)	0.166		1.21 (1.02, 1.43)	0.032		1.49 (1.23, 1.82)	<0.001

38–47 years	0.84 (0.63, 1.13)	0.246		1.10 (0.89, 1.37)	0.367		0.80 (0.61, 1.04)	0.090

48–57 years	1.90 (1.40, 2.58)	<0.001		1.18 (0.91, 1.52)	0.206		1.03 (0.76, 1.39)	0.848

58+ years	0.35 (0.19, 0.65)	0.001		0.96 (0.68, 1.37)	0.828		0.80 (0.52, 1.25)	0.332

**Marital Status**								

Single	***1.00***			***1.00***			***1.00***	

Married	0.68 (0.56, 0.84)	<0.001		1.42 (1.20, 1.68)	<0.001		1.69 (1.36, 2.10)	<0.001

Divorced/Widowed	1.12 (0.71, 1.78)	0.626		1.71 (1.15, 2.53)	0.008		2.43 (1.56, 3.76)	<0.001

**Working Status**								

Working (Full time)	***1.00***			***1.00***			***1.00***	

Working (Part time)	1.23 (0.97, 1.56)	0.089		0.74 (0.61, 0.89)	0.002		0.88 (0.71, 1.10)	0.281

No working/Student	0.72 (0.59, 0.89)	0.002		0.40 (0.33, 0.47)	<0.001		0.27 (0.22, 0.34)	<0.001

***Household factor***								

**Living Arrangement**								

Living with Family	***1.00***			***1.00***			***1.00***	

Living Alone	1.27 (1.04, 1.54)	0.019		1.91 (1.61, 2.28)	<0.001		2.22 (1.80, 2.74)	<0.001

Shared accommodation/Living with Flatmates	1.34 (1.02, 1.77)	0.039		1.77 (1.37, 2.29)	<0.001		1.68 (1.20, 2.35)	0.002

**Household Size**								

1–2 People	***1.00***			***1.00***			***1.00***	

3–4 People	0.55 (0.45, 0.78)	<0.001		0.72 (0.59, 0.87)	0.001		0.60 (0.48, 0.75)	<0.001

**Have you been tested for COVID-19?**								

No	***1.00***			***1.00***			***1.00***	

Yes, I Tested Negative	1.85 (1.38, 2.49)	<0.001		2.52 (1.94, 3.26)	<0.001		2.24 (1.67, 3.02)	<0.001

Yes, I Tested Positive	1.48 (0.87, 2.54)	0.152		2.08 (1.31, 3.28)	0.002		1.92 (1.17, 3.16)	0.010

***Compliance with Public health measures***								

**Are You Currently in Self-Quarantine Since Past Seven Days?**								

No	***1.00***			***1.00***			***1.00***	

Yes	0.77 (0.66, 0.91)	0.002		1.41 (1.21, 1.64)	<0.001		1.89 (1.53, 2.33)	<0.001

**Avoid Public Transport**								

Yes	***1.00***			***1.00***			***1.00***	

No	0.91 (0.62, 1.33)	0.626		2.57 (1.86, 3.56)	<0.001		2.52 (1.70, 3.73)	<0.001

**Avoid Shaking Hands**								

Yes	***1.00***			***1.00***			***1.00***	

No	0.69 (0.48, 1.00)	0.051		1.08 (0.78, 1.48)	0.651		1.76 (1.20, 2.56)	0.003

**Wearing Face Mask when going out**								

Yes	***1.00***			***1.00***			***1.00***	

No	0.92 (0.66, 1.28)	0.634		1.28 (0.97, 1.71)	0.081		1.39 (0.99, 1.96)	0.057

**Avoid Large Gatherings**								

Yes	***1.00***			***1.00***			***1.00***	

No	1.53 (1.10, 2.12)	0.011		0.62 (0.46, 0.82)	0.001		0.67 (0.48, 0.95)	0.023

**Advocating People About the Health Risk Related to COVID-19**								

Yes	***1.00***			***1.00***			***1.00***	

No	0.76 (0.61, 0.95)	0.017		0.50 (0.41, 0.62)	<0.001		0.56 (0.43, 0.73)	<0.001

**Health Condition experienced in the Last Week**								

**Fever**								

No	***1.00***			***1.00***			***1.00***	

Yes	1.25 (0.99, 1.57)	0.051		0.90 (0.74, 1.10)	0.290		1.37 (1.09, 1.72)	0.006

**Chills**								

No	***1.00***			***1.00***			***1.00***	

Yes	1.32 (1.01, 1.72)	0.041		2.05 (1.65, 2.56)	<0.001		2.19 (1.72, 2.78)	<0.001

**Headache**								

No	***1.00***			***1.00***			***1.00***	

Yes	1.15 (0.98, 1.36)	0.089		1.43 (1.25, 1.64)	<0.001		1.13 (0.96, 1.35)	0.144

**Cough**								

No	***1.00***			***1.00***			***1.00***	

Yes	2.55 (2.14, 3.03)	<0.001		2.45 (2.10, 2.86)	<0.001		1.68 (1.39, 2.02)	<0.001

**Breathing Difficulty**								

No	***1.00***			***1.00***			***1.00***	

Yes	3.29 (2.48, 4.35)	<0.001		3.15 (2.47, 4.03)	<0.001		4.43 (3.41, 5.76)	<0.001

**Dizziness**								

No	***1.00***			***1.00***			***1.00***	

Yes	1.92 (1.53, 2.40)	<0.001		2.42 (1.99, 2.93)	<0.001		1.86 (1.49, 2.32)	0.001

**Sore Throat**								

No	***1.00***			***1.00***			***1.00***	

Yes	1.84 (1.48, 2.27)	<0.001		2.26 (1.89, 2.70)	<0.001		2.15 (1.75, 2.65)	<0.001


Adjusted odd ratios (aOR); 95% confidence intervals (CI).

### Factors associated with symptoms of anxiety

**Figure 3:**
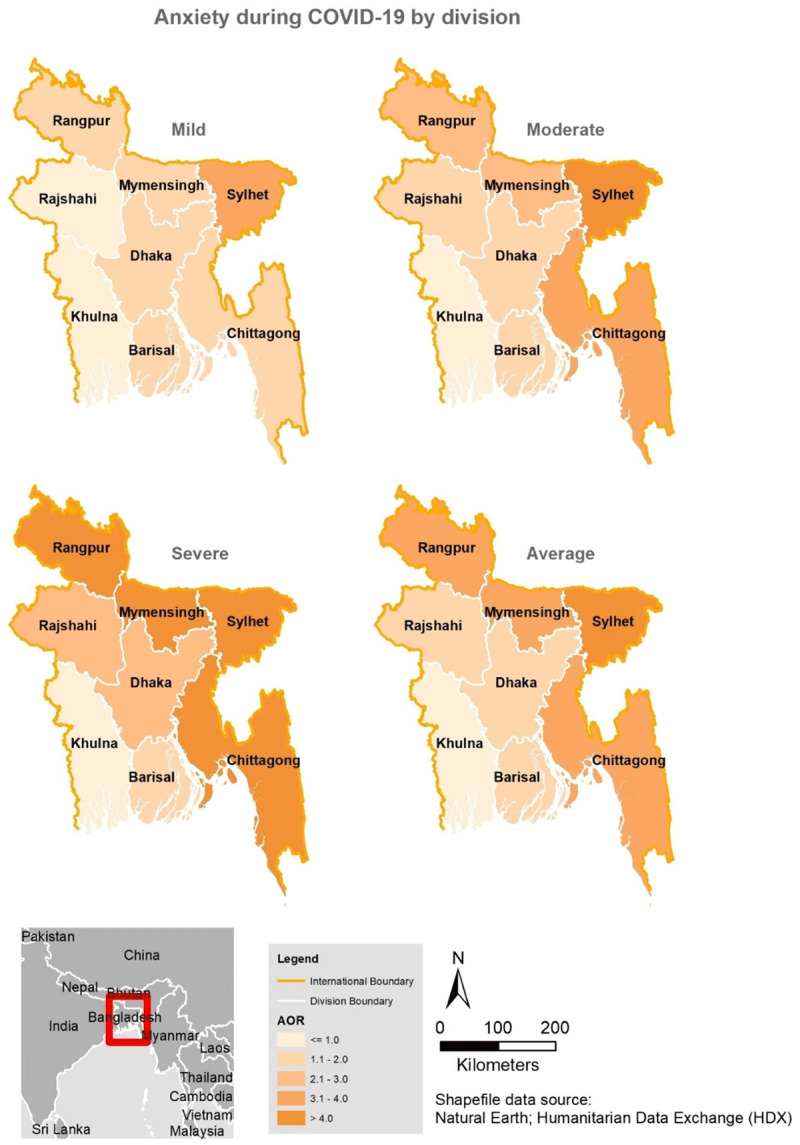
Spatial distribution of anxiety during COVID-19 in Bangladesh by division.

***Table 3*** presents the multivariate analysis of factors associated with anxiety. Females, those who were divorced or widowed, those who lived in shared accommodation during the lockdown, individuals who experienced self-quarantine, and those who experienced fever and cough a couple of weeks before data collection were more likely to experience mild-severe symptoms of anxiety, while older participants experienced a lower risk of anxiety at all levels. The distribution by region (***Figure 3***) revealed greater than a threefold increase in the odds of feeling severely anxious among respondents residing in four regions. Compared to those from Khulna division, participants who lived in other divisions, particularly Chittagong [aOR, 6.26, 95%CI, 4.40–8.90], Mymensing [aOR 5.26, 95%CI, 3.04–9.12], and Rangpur [aOR, 5.86, 95%CI, 3.64–9.42] were more likely to feel severely anxious (see ***Figure 3***) as well as those who lived alone [aOR, 1.70, 95%CI, 1.35–2.16] during the lockdown. Those with confirmed or suspected cases of COVID-19 were about two times more likely to experience severe symptoms of anxiety compared to those who were not tested. Other symptoms of COVID-19 were also significantly associated with some degree of anxiety in this study.

**Table 3:** Factors associated with mild, moderate, and severe anxiety during COVID-19 in Bangladesh.


VARIABLES	MILD			MODERATE			SEVERE	
		
	aOR	P-VALUE		aOR	P-VALUE		aOR	P-VALUE

***Demography***								

**Division of Living**								

Khulna	***1.00***			***1.00***			***1.00***	

Chittagong	1.38 (0.91, 2.09)	0.124		3.28 (2.23, 4.83)	<0.001		6.26 (4.40, 8.90)	<0.001

Mymensing	1.57 (0.84, 2.92)	0.159		2.30 (1.24, 4.26)	0.008		5.26 (3.04, 9.12)	<0.001

Rajshahi	0.65 (0.44, 0.95)	0.026		1.11 (0.77, 1.60)	0.582		2.26 (1.68, 3.04)	<0.001

Barisal	1.25 (0.92, 1.69)	0.149		1.07 (0.76, 1.50)	0.697		1.60 (1.21, 2.11)	0.001

Rangpur	1.19 (0.67, 2.11)	0.561		2.17 (1.26, 3.75)	0.005		5.86 (3.64, 9.42)	<0.001

Sylhet	3.32 (0.38, 28.84)	0.277		20.25 (2.71, 151.42)	<0.001		38.54 (5.30, 280.02)	<0.001

Dhaka	1.13 (0.93, 1.38)	0.233		1.70 (1.38, 2.09)	<0.001		2.91 (2.44, 3.47)	<0.001

**Gender**								

Male	***1.00***			***1.00***			***1.00***	

Female	1.20 (1.01, 1.43)	0.039		1.53 (1.29, 1.82)	<0.001		1.91 (1.65, 2.21)	<0.001

**Level of Education**								

Postgraduate/Post Doctorate	***1.00***			***1.00***			***1.00***	

Graduate	1.10 (0.89, 1.36)	0.363		1.19 (0.97, 1.47)	<0.001		1.51 (1.26, 1.81)	<0.001

HSC or Equivalent	0.75 (0.59, 0.95)	0.016		1.06 (0.84, 1.34)	0.608		1.78 (1.46, 2.16)	<0.001

SSC or Equivalent	0.96 (0.69, 1.34)	0.811		0.88 (0.62, 1.25)	0.465		2.34 (1.78, 3.07)	<0.001

Under SSC	2.13 (1.38, 3.28)	0.001		1.45 (0.90, 2.33)	0.124		3.37 (2.30, 4.94)	<0.001

**Age in category**								

18–27 years	***1.00***			***1.00***			***1.00***	

28–37 years	0.46 (0.35, 0.60)	<0.001		0.60 (0.46, 0.78)	<0.001		0.45 (0.36, 0.56)	<0.001

38–47 years	0.39 (0.28, 0.54)	<0.001		0.30 (0.21, 0.42)	<0.001		0.27 (0.21, 0.36)	<0.001

48–57 years	0.19 (0.12, 0.28)	<0.001		0.30 (0.20, 0.44)	<0.001		0.31 (0.23, 0.42)	<0.001

58+ years	0.10 (0.056, 0.18)	<0.001		0.17 (0.10. 0.29)	<0.001		0.18 (0.12, 0.26)	<0.001

**Marital Status**								

Single	***1.00***			***1.00***			***1.00***	

Married	0.76 (0.58, 0.98)	0.037		0.80 (0.62, 1.03)	0.088		1.09 (0.88, 1.35)	0.433

Divorced/Widowed	4.98 (2.22, 11.13)	<0.001		6.38 (2.94, 13.89)	<0.001		6.00 (2.88, 12.50)	<0.001

**Working Status**								

Working (Full time)	***1.00***			***1.00***			***1.00***	

Working (Part time)	0.90 (0.66, 1.23)	0.505		0.90 (0.67, 1.23)	0.520		0.79 (0.61, 1.01)	0.058

No working/Student	0.78 (0.61, 1.01)	0.060		0.96 (0.74, 1.24)	0.761		0.51 (0.41, 0.63)	<0.001

***Household factor***								

**Living Arrangement**								

Living with Family	***1.00***			***1.00***			***1.00***	

Living Alone	0.83 (0.62, 1.12)	0.227		1.13 (0.86, 1.45)	0.366		1.70 (1.35, 2.16)	<0.001

Shared accommodation/Living with Flatmates	1.87 (1.19, 2.92)	0.006		2.10 (1.34, 3.29)	0.001		2.42 (1.60, 3.64)	<0.001

**Household Size**								

1–2 People	***1.00***			***1.00***			***1.00***	

3–4 People	0.61 (0.42, 0.88)	0.008		0.63 (0.45, 0.90)	0.011		0.37 (0.27, 0.50)	<0.001

**Have you been tested for COVID-19?**								

No	***1.00***			***1.00***			***1.00***	

Yes, I Tested Negative	1.02 (0.65, 1.61)	0.932		1.38 (0.92, 2.07)	0.120		1.85 (1.31, 2.63)	0.001

Yes, I Tested Positive	1.45 (0.56, 3.74)	0.439		2.44 (1.05, 5.68)	0.038		2.31 (1.05, 5.09)	0.038

***Compliance with Public health measures***								

**Are You Currently in Self-Quarantine Since Past Seven Days?**								

No	***1.00***			***1.00***			***1.00***	

Yes	2.27 (1.81, 2.86)	<0.001		1.53 (1.23, 1.89)	<0.001		1.44 (1.20, 1.73)	<0.001

**Avoid Public Transport**								

Yes	***1.00***			***1.00***			***1.00***	

No	0.40 (0.21, 0.74)	0.004		0.74 (0.42, 1.32)	0.306		1.49 (0.88, 2.52)	0.135

**Avoid Shaking Hands**								

Yes	***1.00***			***1.00***			***1.00***	

No	0.52 (0.28, 0.94)	0.030		0.48 (0.28, 0.85)	0.011		0.81 (0.49, 1.34)	0.411

**Wearing Face Mask when going out**								

Yes	***1.00***			***1.00***			***1.00***	

No	2.34 (1.40, 3.94)	0.001		1.15 (0.71, 1.87)	0.574		1.82 (1.17, 2.81)	0.007

**Avoid Large Gatherings**								

Yes	***1.00***			***1.00***			***1.00***	

No	1.20 (0.70, 2.05)	0.500		0.94 (0.57, 1.56)	0.819		0.48 (0.30, 0.76)	0.002

**Advocating People About the Health Risk Related to COVID-19**								

Yes	***1.00***			***1.00***			***1.00***	

No	0.62 (0.43, 0.89)	0.009		0.58 (0.42, 0.83)	0.003		0.36 (0.26, 0.50)	<0.001

**Health Condition experienced in the Last Week**								

**Fever**								

No	***1.00***			***1.00***			***1.00***	

Yes	2.07 (1.35, 3.17)	0.001		1.92 (1.27, 2.89)	0.002		2.80 (1.92, 4.08)	<0.001

**Chills**								

No	***1.00***			***1.00***			***1.00***	

Yes	1.32 (0.78, 2.25)	0.299		2.75 (1.74, 4.35)	<0.001		4.63 (3.05, 7.02)	<0.001

**Headache**								

No	***1.00***			***1.00***			***1.00***	

Yes	2.54 (1.98, 3.27)	<0.001		2.43 (1.90, 3.10)	<0.001		2.17 (1.74, 2.69)	0.144

**Cough**								

No	***1.00***			***1.00***			***1.00***	

Yes	2.17 (1.62, 2.90)	<0.001		2.51 (1.90, 3.31)	<0.001		2.81 (2.18, 3.63)	<0.001

**Breathing Difficulty**								

No	***1.00***			***1.00***			***1.00***	

Yes	0.63 (0.41, 0.97)	0.038		1.03 (0.71, 1.50)	0.878		1.67 (1.21, 3.30)	0.002

**Dizziness**								

No	***1.00***			***1.00***			***1.00***	

Yes	0.88 (0.63, 1.23)	0.458		1.21 (0.89, 1.65)	0.215		1.21 (0.92, 1.59)	0.170

**Sore Throat**								

No	***1.00***			***1.00***			***1.00***	

Yes	0.86 (0.60, 1.22)	0.390		2.13 (1.57, 2.91)	<0.001		1.88 (1.42, 2.49)	<0.001


Adjusted odd ratios (aOR); 95% confidence intervals (CI).

### Factors associated with symptoms of stress

**Figure 4:**
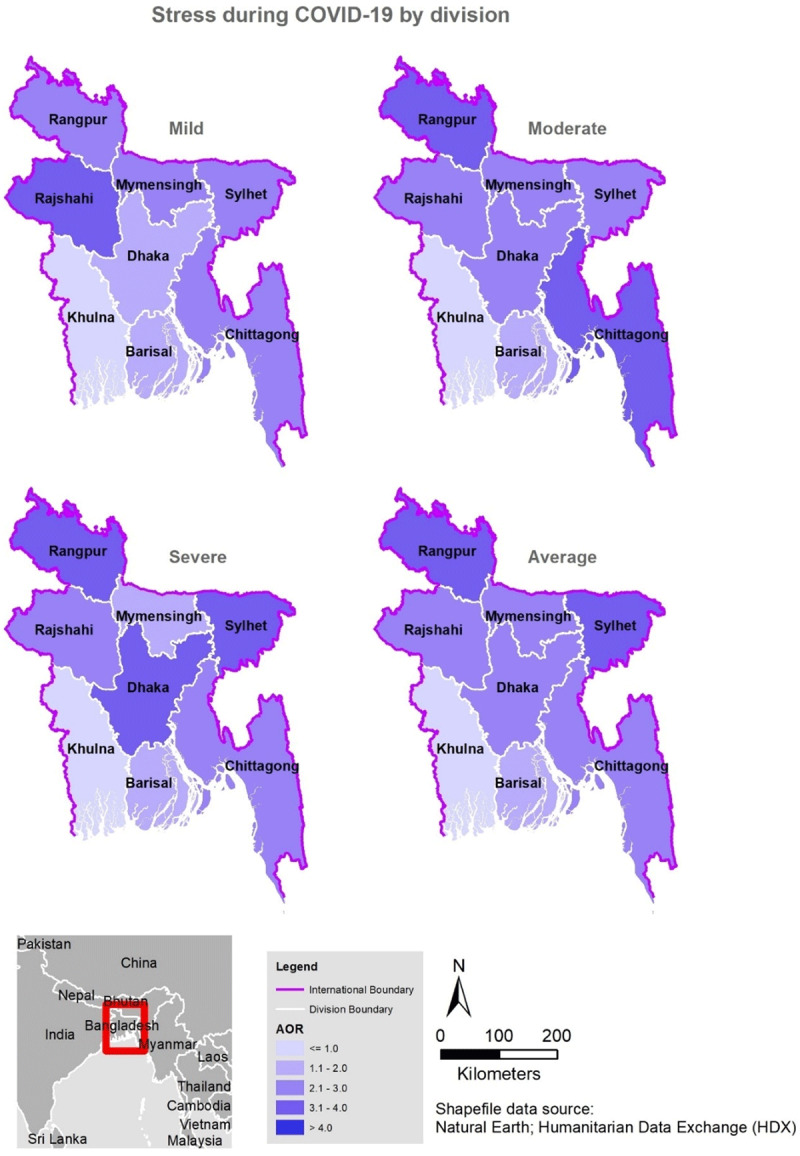
Spatial distribution of stress during COVID-19 in Bangladesh by division.

The factors associated with stress in the multivariate analysis are shown in ***Table 4***, while the regional distribution is shown in ***Figure 4***. Those who lived in Dhaka, Rangpur, and Silhet experienced severe levels of stress during the pandemic (***Figure 4***). Females, those living outside of Khulna division, participants with lower than postgraduate education, those older than 27years, were married, lived alone, and had experienced self-quarantine and any of the health symptoms in this study (except for fever), were more likely to experience symptoms of stress at all levels. Participants who were divorced/widowed [aOR, 1.54, 95%CI, 1.06–2.24], those who lived in shared accommodations [aOR, 2.26, 95%CI, 1.69–3.02], as well as individuals who failed to comply with the precautionary measures advising people to avoid traveling by public transport and/or shake hands had a remarkably higher odds of moderate (aOR, 4.02, 95%CI, 2.74–5.91) and severe stress symptoms (aOR, 1.63, 95%CI, 1.15–2.30], while severe stress symptoms were also found among those who failed to wear face masks when going out [aOR, 1.64, 95%CI, 1.19–2.25].

**Table 4:** Factors associated with mild, moderate, and severe stress during COVID-19 in Bangladesh.


VARIABLES	MILD			MODERATE			SEVERE	
		
	aOR	P-VALUE		aOR	P-VALUE		aOR	P-VALUE

***Demography***								

**Division of Living**								

Khulna	***1.00***			***1.00***			***1.00***	

Chittagong	2.10 (1.61, 2.74)	<0.001		3.70 (2.92, 4.68)	<0.001		2.87 (2.17, 3.78)	<0.001

Mymensing	2.18 (1.44, 3.29)	<0.001		2.10 (1.44, 3.08)	<0.001		1.85 (1.18, 2.89)	0.007

Rajshahi	3.16 (2.36, 4.23)	<0.001		2.72 (2.05, 3.60)	<0.001		2.98 (2.18, 4.06)	<0.001

Barisal	1.25 (0.91, 1.71)	0.168		1.82 (1.38, 2.39)	<0.001		1.36 (0.97, 1.89)	0.074

Rangpur	2.51 (1.76, 3.58)	<0.001		3.73 (2.723, 5.11	<0.001		3.21 (2.23, 4.61)	<0.001

Sylhet	2.86 (1.81, 4.52)	<0.001		2.71 (1.72, 4.37)	<0.001		3.77 (2.32, 6.13)	<0.001

Dhaka	1.82 (1.49, 2.23)	<0.001		2.42 (2.00, 2.91)	<0.001		3.07 (2.48, 3.80)	<0.001

**Gender**								

Male	***1.00***			***1.00***			***1.00***	

Female	1.62 (1.42, 1.87)	<0.001		1.54 (1.37, 1.74)	<0.001		1.82 (1.58, 2.09)	<0.001

**Level of Education**								

Postgraduate/Post Doctorate	***1.00***			***1.00***			***1.00***	

Graduate	1.85 (1.54, 2.22)	<0.001		2.11 (1.80, 2.47)	<0.001		1.89 (1.58, 2.27)	<0.001

HSC or Equivalent	1.69 (1.38, 2.06)	<0.001		1.92 (1.62, 2.28)	<0.001		1.98 (1.64, 2.39)	<0.001

SSC or Equivalent	2.61 (2.02, 3.37)	<0.001		2.56 (2.03, 3.22)	<0.001		3.09 (2.41, 3.94)	<0.001

Under SSC	1.72 (1.24, 2.40)	0.001		1.61 (1.20, 2.17)	0.002		2.43 (1.79, 3.30)	<0.001

**Age in category**								

18–27 years	***1.00***			***1.00***			***1.00***	

28–37 years	1.72 (1.42, 2.09)	<0.001		1.49 (1.27, 1.76)	<0.001		1.61 (1.35, 1.92)	<0.001

38–47 years	1.96 (1.54, 2.50)	<0.001		1.49 (1.21, 1.84)	<0.001		1.42 (1.13, 1.80)	0.003

48–57 years	1.16 (0.85, 1.60)	0.345		1.15 (0.89, 1.49)	0.275		1..95 (1.51, 2.51)	<0.001

58+ years	5.31 (3.63, 7.76)	<0.001		2.25 (1.53, 3.33)	<0.001		2.83 (1.87, 4.28)	<0.001

**Marital Status**								

Single	***1.00***			***1.00***			***1.00***	

Married	1.72 (1.41, 2.11)	<0.001		1.43 (1.20, 1.71)	<0.001		2.05 (1.67, 2.51)	<0.001

Divorced/Widowed	1.97 (1.38, 2.82)	<0.001		1.31 (0.94, 1.83)	0.106		1.54 (1.06, 2.24)	0.024

**Working Status**								

Working (Full time)	***1.00***			***1.00***			***1.00***	

Working (Part time)	1.11 (0.90, 1.38)	0.329		0.91 (0.76, 1.10)	0.324		0.68 (0.56, 0.84)	<0.001

No working/Student	0.96 (0.79, 1.17)	0.714		0.36 (0.31, 0.43)	<0.001		0.27 (0.22, 0.34)	<0.001

***Household factor***								

**Living Arrangement**								

Living with Family	***1.00***			***1.00***			***1.00***	

Living Alone	1.60 (1.33, 1.93)	<0.001		1.67 (1.41, 1.98)	<0.001		1.71 (1.40, 2.08)	<0.001

Shared accommodation/Living with Flatmates	1.02 (0.75, 1.40)	0.883		1.59 (1.23, 2.06)	<0.001		2.26 (1.69, 3.02)	<0.001

**Household Size**								

1–2 People	***1.00***			***1.00***			***1.00***	

3–4 People	0.76 (0.62, 0.93)	0.007		0.74 (0.61, 0.88)	0.001		0.58 (0.47, 0.70)	<0.001

**Have you been tested for COVID-19?**								

No	***1.00***			***1.00***			***1.00***	

Yes, I Tested Negative	1.57 (1.23, 2.01)	<0.001		1.77 (1.42, 2.21)	<0.001		1.22 (0.94, 1.57)	0.136

Yes, I Tested Positive	1.38 (0.95, 2.01)	0.095		1.42 (1.01, 1.99)	0.046		1.10 (0.75, 1.60)	0.636

***Compliance with Public health measures***								

**Have You Enforced Protective Measures Inside Your Home to Protect Yourself and Your Family from COVID 19?**								

***Yes***	***1.00***			***1.00***			***1.00***	

***No***	1.08 (0.85, 1.38)	0.540		1.18 (0.94, 1.47)	0.148		1.28 (1.00, 1.65)	0.049

**Are You Currently in Self-Quarantine Since Past Seven Days?**								

No	***1.00***			***1.00***			***1.00***	

Yes	1.28 (1.07, 1.53)	0.007		2.09 (1.75, 2.49)	<0.001		1.92 (1.55, 2.38)	<0.001

**Avoid Public Transport**								

Yes	***1.00***			***1.00***			***1.00***	

No	1.08 (0.80, 1.47)	0.601		1.60 (1.20, 2.13)	0.001		4.02 (2.74, 5.91)	<0.001

**Avoid Shaking Hands**								

Yes	***1.00***			***1.00***			***1.00***	

No	0.94 (0.70, 1.27)	0.700		0.68 (0.52, 0.90)	0.007		1.63 (1.15, 2.30)	0.006

**Wearing Face Mask when going out**								

Yes	***1.00***			***1.00***			***1.00***	

No	1.25 (0.95, 1.64)	0.107		1.15 (0.89, 1.48)	0.291		1.64 (1.19, 2.25)	0.002

**Avoid Large Gatherings**								

Yes	***1.00***			***1.00***			***1.00***	

No	1.12 (0.86, 1.47)	0.398		1.72 (1.35, 2.19)	<0.001		1.18 (0.88, 1.58)	0.257

**Advocating People About the Health Risk Related to COVID-19**								

Yes	***1.00***			***1.00***			***1.00***	

No	0.55 (0.45, 0.68)	<0.001		0.99 (0.81, 1.21)	0.916		0.66 (0.51, 0.84)	0.001

**Health Condition experienced in the Last Week**								

**Fever**								

No	***1.00***			***1.00***			***1.00***	

Yes	0.96 (0.78, 1.17)	0.663		1.11 (0.93, 1.32)	0.252		1.07 (0.88, 1.31)	0.509

**Chills**								

No	***1.00***			***1.00***			***1.00***	

Yes	1.99 (1.62, 2.44)	<0.001		2.07 (1.72, 2.49)	<0.001		2.01 (1.65, 2.46)	<0.001

**Headache**								

No	***1.00***			***1.00***			***1.00***	

Yes	1.26 (1.08, 1.48)	0.004		1.63 (1.42, 1.86)	<0.001		1.50 (1.29, 1.75)	<0.001

**Cough**								

No	***1.00***			***1.00***			***1.00***	

Yes	1.27 (1.08, 1.50)	0.004		1.79 (1.56, 2.07)	<0.001		1.32 (1.12, 1.55)	0.001

**Breathing Difficulty**								

No	***1.00***			***1.00***			***1.00***	

Yes	2.14 (1.74, 2.62)	<0.001		1.73 (1.44, 2.08)	<0.001		2.18 (1.79, 2.66)	<0.001

**Dizziness**								

No	***1.00***			***1.00***			***1.00***	

Yes	1.45 (1.20, 1.76)	<0.001		1.91 (1.63, 2.25)	<0.001		1.50 (1.25, 1.80)	<0.001

**Sore Throat**								

No	***1.00***			***1.00***			***1.00***	

Yes	1.33 (1.11, 1.60)	0.002		1.38 (1.18, 1.61)	<0.001		1.39 (1.16, 1.66)	<0.001


Adjusted odd ratios (aOR); 95% confidence intervals (CI).

## Discussion

This cross-sectional survey used an online survey to assess the prevalence and associated factors of mental health symptoms related to the COVID-19 pandemic in Bangladesh. The study utilized internationally recognized scales and found a high prevalence of mental health symptoms during the pandemic, particularly feeling anxious and depressed, and about half of the respondents experienced stress. Respondents who reported these mental health symptoms during the pandemic were more likely to be females, be married, have lower education, face various factors related to accommodation and living arrangements, and experience COVID-19 related health symptoms. In addition, respondents who were tested for COVID-19, those who traveled via public transport, and people who practiced self-quarantine self-reported a higher prevalence of depression, anxiety, and stress in this study.

In this study, the prevalence of mental health symptoms was higher than previous reports from the United Kingdom [26] and Iran [27] during the COVID-19 pandemic further confirming the high burden of the disease in this region [14]. Similar high prevalence of depression, anxiety, and psychological distress have been reported in other countries during a pandemic including Ethiopia [28] and Australia, particularly during the highly infectious equine influenza in 2007 [29]. Although the higher prevalence of mental health symptoms found in this study may be related to the methods used in assessing the mental health symptoms, it is an indication of an unmet need in the country’s health care system that is fueled by the pandemic and lack of psychiatrists to fulfil these needs [30].

Previous epidemiological studies reported that women were at a higher risk of depression [31] than men and were more vulnerable to stress and post-traumatic stress disorder than men [32]. These findings were corroborated in recent studies where the prevalence of anxiety, depression, and stress during the COVID-19 pandemic was significantly higher among women than men [33,34,35]. Compared with the previous studies, the present study used a larger sample size to confirm that women in Bangladesh experienced significantly higher mental health symptoms than men, and this could be due to the higher representation of women in various industries like retail, manufacturing, healthcare, and service, which were affected by the current pandemic. With the uneven effects in the employment sector, there are suggestions that women were more likely to experience psychological and mental health problems when faced with depression, anxiety, and stress [36].

Older people had a higher risk of COVID-19 infection and mortality [37]; however, the results of existing studies found higher levels of anxiety, depression, and stress among the younger population, particularly those aged 21–40 years [11,38]. That age was associated with the participants’ report of depression, anxiety, and stress in this study may be attributed to the fact that people in this age group are more concerned over the future consequences and economic challenges caused by the pandemic, as they are the key actors in society’s workforce and are, therefore, mostly affected by fear of joblessness and business closures [39]. Some researchers have argued that greater anxiety among young people may be related to their greater access to information through social media, which can also cause severe stress [40,41]. And people become stressed and feel anxious when information from public health experts is unreliable or delivered incorrectly and as such could create confusion regarding the practice of self-quarantine or other public health measures put in place to control the spread of a pandemic [42].

This study found a significant association between level of education and mental health symptoms during the pandemic, which was consistent with the reports of worse mental status among the higher socio-economic class in Bangladesh [43] and among educated adolescents living in urban Dhaka [44]. Similarly, a study conducted in China at the initial stage of the COVID-19 outbreak [45] found that those with no formal education were more likely to report depression during the epidemic. Other studies have also reported significant associations between lower level of education and anxiety and depression levels [26,28]. In contrast to these studies, we found that during the COVID-19 pandemic, respondents in Bangladesh who had a higher level of education reported higher levels of anxiety, depression, and stress [35,46]: 59.75% of respondents of our study had a university level of education in comparison to respondents of the previous studies. Even some recent studies revealed similar findings to our study [47].

Similar to the findings of the present study, some authors in China [45,48] reported higher levels of anxiety among participants who had at least one family member, relative, or friend with COVID-19. That more than two thirds of the participants had at least one symptom of COVID-19 in the weeks preceding data collection and some tested positive to the virus may have contributed to the heightened prevalence of mental health symptoms reported in this study. Similar to a study in the USA [49], we found that being separated or widowed/divorced increased the likelihood of the respondents experiencing symptoms of depression during the pandemic.

During the early outbreak of COVID-19 in Bangladesh, people who came into contact with the infection were asked to isolate themselves at home or in a dedicated quarantine facility [1]. These isolation and quarantine measures increased the likelihood of mental health symptoms as more than two thirds of the study participants who practiced these measures reported mental health issues. Such negative psychological effects of the preventive measures have been reported previously during the SARS outbreak, with an increase in the levels of post-traumatic stress symptoms, confusion, and anger among residents [41]. Other studies found that self-quarantine and isolation measures, as well as employment uncertainty and the rapid spread of COVID-19 related misinformation were associated with peoples experience of psychological problems during the pandemic [40,50,51]. Due to self-quarantine measures and fear related to the spread of COVID-19, other persistent mental health disorders like anxiety, emotional disruption, and exhaustion, depression, anger, irritability, insomnia, and stress can be developed among the population [52]. Moreover, the longer the quarantine or self-isolation, the more detrimental these outcomes can become [53].

### Limitations and strengths

This study had some limitations. First, it was limited in scope. Many of the participants (50%) were from the capital city of Dhaka division, limiting the generalization of our findings to rural regions. Second, the study was carried out during the COVID-19 lockdown period and lacked longitudinal follow-up. The analysis of the periodic state of individuals may not reflect the psychological state, which changes with time and with alterations in one’s surrounding environment. Because of the increasingly arduous situation and the fear of the second wave, the mental health symptoms of residents could become more severe. Thus, the long-term psychological implications of this population are worth further investigation. Third, due to ethical requirements on anonymity and confidentiality, we were not allowed to collect contact details and personal information from the respondents. As a result, we could not conduct a prospective study that would provide a concrete finding to support the need for a focused public health initiative. Fourth, the study was not able to distinguish between pre-existing mental health symptoms and new symptoms. Fifth, there was an oversampling of a particular network of peers (e.g., students), which may lead to selection bias. However, a large percentage of respondents who were within 40 years old consisted of students. They were exposed to higher mental health symptoms due to the temporary shutdown of educational institutions, disbandment of social gatherings, and pressure from having to attend classes online [54]. Sixth, the self-reported levels of psychological impact, anxiety, depression, and stress may not always align with assessment by mental health professionals.

Despite these limitations, this study has several strengths. The study provides additional global data to inform understanding of the impact of the COVID-19 pandemic in developing countries. The findings also advance the knowledge of mental health impact during the COVID-19 pandemic in such a region with a struggling health care system by providing potentially helpful data to inform possible public health management strategizing. The findings also shed added light on the impact of COVID-19 on the mental health conditions of the educated people in Bangladesh who are the potential future workforce. An impressive strength of our study is the data volume (>10,000 survey respondents), generally exceeding that of similar studies. In addition, this study used impressive GIS maps to highlight the most affected areas in Bangladesh.

## Conclusions

In this survey study conducted in Bangladesh, respondents reported high rates of symptoms of depression, anxiety, and distress while non-compliance with public health measures increased the risk of mental health outcomes. Protecting the Bangladesh population is an important component of public health measures for addressing the COVID-19 pandemic. Special interventions to promote mental well-being in Bangladesh communities exposed to COVID-19 need to be immediately implemented, with women, married people, less-educated people, and those that were tested for COVID-19 requiring particular attention. To further close the gap in the relationship and improve the mental health and well-being of the Bangladeshi people, alternative ways of communication, such as the use of internet video calls [55], should be promoted during similar situations.

## Data Accessibility Statement

Our data are included in the manuscript, and raw data can be released on reasonable request.

## Additional Files

The additional files for this article can be found as follows:

10.5334/aogh.3269.s1 Supplementary Table 1. Sample of survey items and their test of reliability.

10.5334/aogh.3269.s2 Supplementary Table 2. A survey questionnaire on Mental health and psychological impact of the coronavirus disease 2019 (COVID-19) outbreak: A cross-sectional study on Bangladeshi People.
