# Supplementary material for: Prevalence and Factors Associated with Mental Health Impact of COVID-19 Pandemic in Bangladesh: A Survey-Based Cross-Sectional Study
Source: Ann Glob Health. 2021 Apr 26;87(1):43. doi: 10.5334/aogh.3269 (PMC8086734; doi:10.5334/aogh.3269)
Supplement: Supplementary Table 1. — Sample of survey items and their test of reliability. [file agh-87-1-3269-s1.pdf]

## Supplementary Table 1: Sample of survey items and their test of reliability

| Did not apply to me at all (0) | Applied to me to some degree or some of the time (1) | Applied to me to a considerable degree or most of the time (2) | Applied to me very much or most of the time (3) |
|---|---|---|---|

| **Depression subscale** | Cronbach's Alpha |
|---|---|
| **3. I couldn’t seem to experience any positive feeling at all** | 0.7618 |
| **5. I found it difficult to work up the initiative to do things** | 0.7661 |
| **10. I felt that I had nothing to look forward to** | 0.7594 |
| **13. I felt downhearted and blue** | 0.7690 |
| **16. I was unable to become enthusiastic about anything** | 0.7650 |
| **17. I felt I wasn’t worth much as a person** | 0.7330 |
| **21. I felt that life was meaningless** | 0.7633 |
| **Anxiety subscale** | Cronbach's Alpha |
| **2. I was aware of dryness of my mouth** | 0.6644 |
| **4. I experienced breathing difficulty (e.g. excessively rapid breathing, breathlessness in the absence of physical exertion)** | 0.6768 |
| **7. I experienced trembling (e.g. in the hands)** | 0.7720 |
| **9. I was worried about situations in which I might panic and make a fool of myself** | 0.6668 |
| **15. I felt I was close to panic** | 0.6648 |
| **19. I was aware of the action of my heart in the absence of physical exertion (e.g. sense of heart rate increase, heart missing a beat)** | 0.6513 |
| **20. I felt scared without any good reason** | 0.6511 |
| **Stress subscale** | Cronbach's Alpha |
| **1. I found it hard to wind down** | 0.8100 |
| **6. I tended to over-react to situations** | 0.7894 |
| **8. I felt that I was using a lot of nervous energy** | 0.7776 |
| **11. I found myself getting agitated** | 0.7877 |
| **12. I found it difficult to relax** | 0.7775 |
| **14. I was intolerant of anything that kept me from getting on with what I was doing** | 0.7822 |
| **18. I felt that I was rather touchy** | 0.7823 |
