# Supplementary material for: Prevalence and Factors Associated with Mental Health Impact of COVID-19 Pandemic in Bangladesh: A Survey-Based Cross-Sectional Study
Source: Ann Glob Health. 2021 Apr 26;87(1):43. doi: 10.5334/aogh.3269 (PMC8086734; doi:10.5334/aogh.3269)
Supplement: Supplementary Table 2. — A survey questionnaire on Mental health and psychological impact of the coronavirus disease 2019 (COVID-19) outbreak: A cross-sectional study on Bangladeshi People. [file agh-87-1-3269-s2.pdf]

Supplementary Table 2

A survey questionnaire on Mental health and psychological impact of the coronavirus disease 2019 (COVID-19) outbreak: A cross-sectional study on Bangladeshi People

Socio-Demographic Profile of the Respondent:

**Name**			
**What is your gender**		Male	1
		Female	2
		Other	88
**Level of study (year)**		Under SSC	1
		SSC or Equivalent	2
		HSC or Equivalent	3
		Graduate	4
		Post Graduate	5
		Doctorate	6
		Post Doctorate	7
		Other	88
**Your Mother's Schooling**		Under SSC	1
		SSC or Equivalent	2
		HSC or Equivalent	3
		Graduate	4
		Post Graduate	5
		Doctorate	6
		Post Doctorate	7
		Other	88

**Your Father's Schooling**		Under SSC	1
		SSC or Equivalent	2
		HSC or Equivalent	3
		Graduate	4
		Post Graduate	5
		Doctorate	6
		Post Doctorate	7
		Other	88
**What is your age?**			##
**Marital Status**		Single	1
		Married	2
		Divorced	3
		Widowed	4
**What is your professional status?**		Student	1
		Working (Full Time)	2
		Working (Part-Time	3
		None	4
**Living Arrangement**		Living Alone	1
		Living with Family	2
		Living with flatmates	3
		Others	88
**Which district are you currently living in**			##
**Which district you originally belong to**			##
**Have you been tested for COVID-19?**		Yes, I tested positive	1
		Yes, I tested negative	2
		No	3

**Have you enforced protective measures inside your home to protect yourself and your family from COVID 19?**		Yes	1
		No	2
**Are you currently in Self-Quarantine since past seven days?**		Yes	1
		No	2
**What sort of protective measures have you taken?**		Avoid public transportation	1
		Avoid shaking hands	2
		Wearing face mask	3
		Avoid large gatherings	4
		Advocating people about the health risk related to COVID-19	5
		All of the above	6
		None of the above	7
**What is your household size?**		One person	1
		Two people	2
		Three to five people	3
		Six people or more	4

Physical Health Status (Please respond on your physical health since the past two weeks)

	Yes (1)	No (2)
**Persistent fever >98 ° 'F' for at least one day**		
**Chills**		
**Headache**		
**Cough**		
**Breathing Difficulty**		
**Dizziness**		
**Sore Throat**		
**Persistent fever and cough or difficulty breathing**		

Mental Health Impact of COVID 19:

Please read each statement and circle a number 1, 2, 3 or 4 which shows how much the statement has applied in the past week to you. There are no correct answers or incorrect answers.

	Did not apply to me at all (1)	Applied to me to some degree or some of the time (2)	Applied to me to a considerable degree or most of the time (3)	Applied to me very much or most of the time (4)
I found it hard to wind down				
I was aware of dryness of my mouth				
I couldn't seem to experience any positive feeling at all				
I experienced breathing difficulty (e.g. excessively rapid breathing, breathlessness in the absence of physical exertion)				
I found it difficult to work up the initiative to do things				
I tended to over-react to situations				
I experienced trembling (e.g. in the hands)				
I felt that I was using a lot of nervous energy				
I was worried about situations in which I might panic and make a fool of myself				
I felt that I had nothing to look forward to				
I found myself getting agitated				
I found it difficult to relax				
I felt down-hearted and blue				
I was intolerant of anything that kept me from getting				

**on with what I was doing**				
**I felt I was close to panic**				
**I was unable to become enthusiastic about anything**				
**I felt I wasn't worth much as a person**				
**I felt that I was rather touchy**				
**I was aware of the action of my heart in the absence of physical exertion (e.g. sense of heart rate increase, heart missing a beat)**				
**I felt scared without any good reason**				
**I felt that life was meaningless**				

Thank you for your participation

1. Akram A, Yeasmin M, Khan TEU, Khan MAAZ, Monir MBB, Tarana MN et al. Demographic Characteristics of 1082 Positive Cases of COVID-19 Patients: Experience at National Referral Laboratory of Bangladesh. Bangladesh Journal of Infectious Diseases. 2020:S22-S6.
